# Using rapid diagnostic tests as source of malaria parasite DNA for molecular analyses in the era of declining malaria prevalence

**DOI:** 10.1186/1475-2875-10-6

**Published:** 2011-01-12

**Authors:** Deus S Ishengoma, Sudi Lwitiho, Rashid A Madebe, Nyagonde Nyagonde, Ola Persson, Lasse S Vestergaard, Ib C Bygbjerg, Martha M Lemnge, Michael Alifrangis

**Affiliations:** 1 National Institute for Medical Research, Tanga Centre, Bombo Road, P.O Box 5004, Tanga, Tanzania; 2 Centre for Medical Parasitology at the Department of International Health, Immunology and Microbiology, University of Copenhagen and Department of Infectious Diseases, Copenhagen University Hospital (Rigshospitalet), Denmark

## Abstract

**Background:**

Malaria prevalence has recently declined markedly in many parts of Tanzania and other sub-Saharan African countries due to scaling-up of control interventions including more efficient treatment regimens (e.g. artemisinin-based combination therapy) and insecticide-treated bed nets. Although continued molecular surveillance of malaria parasites is important to early identify emerging anti-malarial drug resistance, it is becoming increasingly difficult to obtain parasite samples from ongoing studies, such as routine drug efficacy trials. To explore other sources of parasite DNA, this study was conducted to examine if sufficient DNA could be successfully extracted from malaria rapid diagnostic tests (RDTs), used and collected as part of routine case management services in health facilities, and thus forming the basis for molecular analyses, surveillance and quality control (QC) testing of RDTs.

**Methods:**

One hyper-parasitaemic blood sample (131,260 asexual parasites/μl) was serially diluted in triplicates with whole blood and blotted on RDTs. DNA was extracted from the RDT dilution series, either immediately or after storage for one month at room temperature. The extracted DNA was amplified using a nested PCR method for *Plasmodium *species detection. Additionally, 165 archived RDTs obtained from ongoing malaria studies were analysed to determine the amplification success and test applicability of RDT for QC testing.

**Results:**

DNA was successfully extracted and amplified from the three sets of RDT dilution series and the minimum detection limit of PCR was <1 asexual parasite/μl. DNA was also successfully amplified from (1) 70/71 (98.6%) archived positive RDTs (RDTs and microscopy positive) (2) 52/63 (82.5%) false negative RDTs (negative by RDTs but positive by microscopy) and (3) 4/24 (16.7%) false positive RDTs (positive by RDTs but negative by microscopy). Finally, 7(100%) negative RDTs (negative by RDTs and microscopy) were also negative by PCR.

**Conclusion:**

This study showed that DNA extracted from archived RDTs can be successfully amplified by PCR and used for detection of malaria parasites. Since Tanzania is planning to introduce RDTs in all health facilities (and possibly also at community level), availability of archived RDTs will provide an alternative source of DNA for genetic studies such as continued surveillance of parasite resistance to anti-malarial drugs. The DNA obtained from RDTs can also be used for QC testing by detecting malaria parasites using PCR in places without facilities for microscopy.

## Background

Malaria has for a long time remained a major cause of morbidity and mortality in most endemic countries particularly in sub-Saharan Africa. However, recent reports have indicated that malaria prevalence has declined drastically in some endemic countries [1-4]. The decline has also been reported in most areas of Tanga region, which was a malaria hyper-endemic area until recently [5] and possibly other parts of Tanzania (National Malaria Control Programme, unpublished data). The declining malaria in Tanga, Tanzania and other countries is possibly attributed to more efficient treatment regimens (using artemisinin combination therapy, ACT) and increased availability and use of insecticide-impregnated bed nets[4].

Following changes of malaria treatment guidelines in Tanzania in 2006, by introducing an ACT containing artemether and lumefantrine (ALu) [6], which is relatively expensive compared to other drugs that were previously used in the country, it is rationally recommended that ALu should be prescribed to only laboratory confirmed cases [7,8]. However, laboratory services are not available in most parts of Tanzania, especially in rural areas and in places with laboratory facilities, the quality of malaria diagnosis is generally poor [9,10] or the results are often ignored by clinicians [11]. Thus, the Tanzanian Ministry of Health and Social Welfare through the National Malaria Control Programme (NMCP) is planning to introduce rapid diagnostic tests (RDTs) in all health facilities to scale-up malaria diagnosis and improve case management.

RDTs detect presence of parasite specific antigens and available RDTs detect either *Plasmodium falciparum *specific histidine rich protein 2 (PfHRP-2), Plasmodium lactate dehydrogenase (pLDH) or aldolase [12-14]. Most of the available tests usually involve blotting a small volume of blood (2-20 μl) on a nitrocellulose strip containing monoclonal antibodies, which react with parasite specific antigens available in the blood of infected patients to give visible, diagnostic and control bands [13]. Despite variability observed in field studies conducted in different settings [15-18], majority of RDTs have high sensitivity and specificity (>90%) at a parasitaemia >100 asexual parasites/μl [13]. The accuracy (sensitivity and specificity) of RDTs is mostly dependent on the parasite species, transmission intensity, parasite density, amount of circulating antigens, local polymorphisms of target antigen and persistence of antigens after treatment [12,14].

Previous genetic studies of malaria parasites have largely relied on extraction of DNA from samples blotted on filter papers [19,20]. The filter papers have usually been easily sampled, for instance as part of drug efficacy trials and general assessments of malaria epidemiology and thus been a reliable source of parasite DNA. However, recently it has become increasingly difficult to obtain parasite samples due to declining malaria in most areas of Tanzania resulting in fewer studies performed, for instance drug efficacy studies. Moreover, the number of parasite positive patients from ongoing trials furthermore limits the sample size available for genetic studies. Despite the decrease in malaria prevalence, it is important for instance to identify emerging drug resistance and to update the molecular anti-malarial resistance maps that are currently being drawn, using sensitive molecular tools.

Alternatively, stained blood smears can be used to recover DNA but the quality of DNA is often low compared to filter papers or whole blood samples [21,22], because the harsh fixation and coloration procedures when preparing a Giemsa-stained smear may hamper extraction and amplification. A study which was conducted in French Guiana showed that parasite DNA could be recovered from fresh and archived RDTs [23]. However, more elaborate studies have not been conducted under different epidemiological and climatic settings to confirm if it is practically possible to recover parasite or human DNA from RDTs (either fresh tests or after extended storage). On the other hand, using RDT widely, even at the community level, is an obvious opportunity to obtain blood for diagnosis as well as storage, and later analysis for detection of resistance gene mutations or co-morbidities. Filter paper is widely used for the latter purpose, but not for diagnosis. Furthermore, the World Health Organization (WHO) recommendations for performing quality assurance (QA) of RDTs requires among other things, that RDTs should be monitored in the field by comparing the results of RDTs and reference microscopy monthly (for 20 blood smears with positive RDT results and 20 with negative results from selected sentinel sites) [24]. However, studies conducted to assess RDT implementation and QC monitoring strategies in malaria endemic areas have revealed that these recommendation cannot be attained due to lack of high quality diagnostic services by microscopy [17,25], and thus different strategies such as using DNA extracted from RDTs for detection of malaria parasites will be needed to achieve the intended goal [17,24,25].

This study was therefore conducted to establish if sufficient parasite DNA could be successfully extracted from different types of fresh and archived RDTs (stored at room temperature in the field/laboratory without any preservatives), and used for molecular analyses. It also explored the potentials of using RDTs as source of DNA for detection of malaria parasites as part of QC testing once the tests will be widely deployed in most health facilities in Tanzania. It is expected that availability of used RDTs will make it possible to obtain sufficient parasite samples and utilize them as sources of DNA for different molecular studies of malaria parasites and supporting the NMCP to perform QC testing of the RDTs that will be deployed in the field.

## Methods

### Study site and sampling

This study was conducted at Amani Biomedical Research Laboratory (AMBRELA) of the National Institute for Medical Research (NIMR) at Tanga Centre in Tanga region, northeastern Tanzania. Archived RDTs were obtained from ongoing studies in Muheza (Interactions between ACT for malaria and anti-retrovirals for HIV/AIDS in co-infected patients in Muheza district, Tanzania - InterACT project) and Korogwe [Passive case detection of fevers using community owned resource persons - CORPs (Ishengoma *et al*, personal communication)] districts of Tanga region, Tanzania.

### Samples and laboratory analysis

A hyper-parasitaemic blood sample (with a parasite density of 131,260 asexual parasites/μl) obtained from a patient attended at Tumaini Health Centre in Tanga was initially diluted to 1,000 asexual parasites/μl with whole blood donated by uninfected donor (with blood group O^+^) and serially diluted (two fold dilutions in triplicate) to 0.01 asexual parasites/μl. About 10 μl of the diluted samples were blotted on RDTs according to manufacturers' instructions (ParaHIT^®^f, Span Diagnostics - Surat, India). One set of the RDTs obtained from the serially diluted samples was immediately used for DNA extraction while two sets were stored at room temperature for a period of one month; one of these had silica preservatives added.

Before DNA extraction, the RDTs cassettes were opened using a sterile scissors and forceps, and the nitrocellulose strips were taken out. The strips were cut into two pieces and the portions containing the sample blotting site were used for extraction. The portions of RDTs that were used for DNA extraction were incubated in 0.5% saponin (in 1X phosphate buffered saline) over-night at room temperature. DNA extraction was performed as for filter papers using Chelex-100 method as previously described [26]. The extraction process was similar for the three sets of RDTs (either immediately or after storage at room temperature for one month, with/without silica preservatives) and DNA was amplified using a nested PCR method for *Plasmodium *species detection according to Snonou *et al *[27]. Amplified products were visualized on 2% agarose gel.

A total of 165 archived RDTs were tested to confirm if DNA can be equally obtained from archived RDTs used for patients' diagnosis under routine clinical settings. The same RDTs were used to assess if they could be utilized as a source of DNA for detection of malaria parasites as part of QC testing of RDTs that will be deployed and used for malaria diagnosis at all levels of health care delivery system in Tanzania. The current QC testing of RDTs requires that 20 blood smears prepared by health workers at selected health facilities from patients with positive and 20 from those with negative RDT results should be examined by experienced technicians at a reference laboratory. However, in health facilities without the capacity for malaria diagnosis by microscopy, health workers cannot prepare blood smears of desirable quality and thus, archived RDTs can potentially be employed as an alternative to microscopy by using DNA extracted from such RDTs to detect malaria parasites. To test if DNA obtained from archived RDTs can be used for detection of molecular markers of anti-malarial drug resistance, DNA from one set of the serial dilutions and 10 archived RDTs were used for detection of parasite genotypes in the *Pfdhfr *gene which is a marker of pyrimethamine resistance using the method described by Alifrangis *et al *[28]

The archived RDT samples included 71 RDTs (positive for both RDT and microcopy) collected at health facilities in Muheza and Korogwe districts and stored without any preservatives at room temperature (temperature ranging from 25 - 34°C). Out of these, 26 RDTs were obtained from patients enrolled in InterACT project in Muheza (on Paracheck Pf^®^, Orchid Biomedical Systems - Mumbai, India) while 45 were from patients attended by CORPs in Korogwe district [on ParaHIT^®^f Span Diagnostics - Surat, India (Ishengoma *et al*, personal communication)]. Other 94 RDT samples (on ParaHIT^®^f), included 24 RDT false positive (positive by RDT but negative by microscopy), 63 false negatives (negative by RDTs but positive by microscopy) and seven negative samples (negative by both RDTs and microcopy) obtained from a cross-sectional survey conducted in May 2010 in four villages in Muheza district where high level of false positive and negative RDT results were observed in previous surveys (Ishengoma *et al*, personal communication). All 165 RDT samples were stored at room temperature in the field or laboratory for a duration of 6 - 95 days (Table 1). DNA extraction and PCR genotyping were performed as in the previous assays mentioned above on individual tubes or 96-well plates depending on the number of samples.

**Table 1:** PCR amplification success of DNA extracted from different types/brand of archived RDTs

Category of RDTs sampled	No. of RDTs	Type/brand of RDT	Mean storage duration in days(range)	PCR results	
				
				Positive (%)	Negative (%)
True positive: RDT +ve/BS +ve	26	Paracheck Pf^®^	28(7-58)	26(100)	0(0)

True positive: RDT +ve/BS +ve	45	ParaHIT ^®^f	47(6-95)	44(97.8)	1(2.2)

False negative: RDT -ve/BS +ve	63	ParaHIT ^®^f	60(61-66)	52(82.5)	11(17.4)*

False positive: RDT +ve/BS -ve	24	ParaHIT ^®^f	60(55-66)	4(16.7)	20(83.3)

True negative: RDT -ve/BS -ve	7	ParaHIT ^®^f	65(63-69)	0(0)	7(100)

RDT = Rapid Diagnostic Test, BS = Blood smear, +ve = positive and -ve = negative

*These samples had low parasite density detected by microscopy (less than 100 asexual parasites/μ; ranged from 16-80 asexual parasites/μl).

## Results

**Figure 1:**
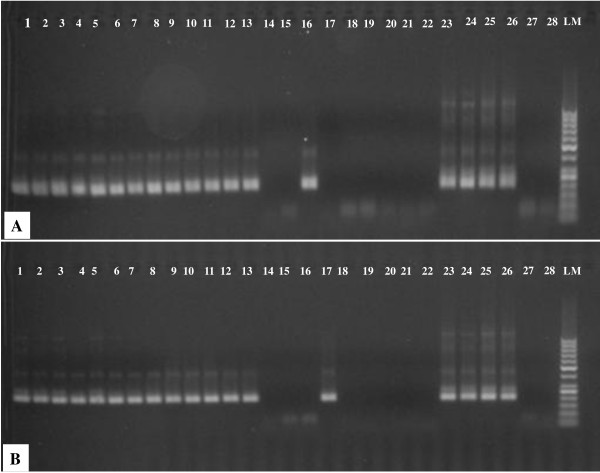
**Pictures of agarose gels showing PCR products of DNA obtained from a hyper-parasitaemic sample which was blotted on RDTs after making two fold serial dilutions (from 1000 to 0.01 asexual parasites/μl) and extracted after storage at room temperature for 30 days with (A) or without (B) silica gels as preservatives**. Lanes 1-22 = DNA from serially diluted samples, 23-26 = positive controls (3D7 strain, 7g8 strain, patient sample with 1000 asexual parasites/μl, field sample on filter paper), 27-28 = negative controls and LM = 50 pb ladder marker.

The minimum parasite detection limit of RDTs for the diluted samples was 10 asexual parasites/μl. DNA was successfully amplified from all of the three sets of RDTs containing the dilution series. RDT samples stored at room temperature for one month either with or without silica gels were amplified equally well, as fresh RDTs analysed immediately after blotting. The minimum parasite detection limit of PCR for all three sets of diluted samples was <1 asexual parasite/μl (Figure 1A and 1B).

**Figure 2:**
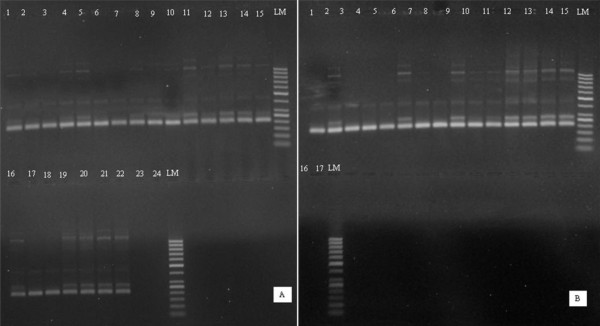
**Selected pictures of agarose gels showing PCR amplification of DNA obtained from 29 of the 71 archived positive RDTs that were used for patients' diagnosis in Muheza and Korogwe districts**. 2A: lanes 1-18 = field samples (16 from InterACT in Muheza in lanes 1-13 and 16-18, and 2 from Korogwe in lanes 14-15), 19-22 = positive controls (3D7strain, FCR3 strain, field sample on filter paper and patient sample with 1000 asexual parasites/μl), 23-24 = negative controls; LM = 50 bp ladder marker 2B: lanes 1-11 = field samples (10 from InterACT in 1-10 and 1 from Korogwe in lane 11); 12-15 = positive controls (3D7 strain, FCR3 strain, field sample on filter paper and patient sample with 1000 asexual parasites/μl), 16-17 = negative controls and LM = 50 bp ladder marker.

Table 1 shows the results of PCR amplification success for DNA extracted from different types of RDTs. Successful amplification was obtained from most of the microscopically positive samples whereby, 70 (98.6%) out of 71 samples, which were positive by both microscopy and RDTs were detected to be positive by PCR (Figure 2A and 2B). Out of these, 26 (100%) RDTs were Paracheck Pf^® ^and 44(97.8%) were ParaHIT^®^f (Table 1). One positive RDT sample (ParaHIT^®^f), which failed to amplify by PCR, had a low parasitaemia detected by microscopy (one asexual parasite/200WBCs equivalent to 40 asexual parasites/μl). Fifty-two (82.5%) of the 63 false negative RDTs (which were negative by RDTs, but positive by microscopy as a gold standard) were positive by PCR. Eleven (17.4%) of the 63 false negative RDT samples which failed to amplify by PCR had low parasite density detected by microscopy (less than 100 asexual parasites/μ; ranged from 16-80 asexual parasites/μl).

Detection of microscopically negative samples was equally successful whereby, 20 out of 24 (83.3%) RDT false positive samples (which were positive by RDTs but negative by microscopy) were confirmed to be negative by PCR. Fourteen out of the 20 RDT false positive samples with negative PCR results were obtained from individuals who had taken anti-malarials (within seven days) before the survey. All seven negative RDTs (negative by both RDTs and microscopy) were also negative by PCR (Table 1). Detection of parasite genotypes for *Pfdhfr *gene as a marker of pyrimethamine resistance using DNA extracted from RDTs was also successful and the results were similar to those observed when DNA was obtained from other sources such as cultured parasites or filter papers.

## Discussion

Previously, malaria has consecutively been shown to be the main cause of morbidity and mortality in Tanga region, as for the rest of Tanzania [29-31]. However, recently a drastic decline in the burden of malaria in this region has been reported [5]; (Ishengoma *et al*, personal communication) and most likely, the decline is apparent in other parts of Tanzania as well (NMCP, Unpublished data). However, because of this current positive trend, recruitment of malaria positive patients for various studies, such as malaria drug efficacy trials has proved to be difficult. Since the region has been known as a hotspot for the emergence of anti-malarial drug resistance in Eastern Africa [32-36], it is of paramount importance to continue the surveillance of in-vivo efficacy of principal anti-malarial drugs in the region. Research-wise, this is furthermore affecting the ability to obtain sufficient number of malaria parasite samples for different molecular studies such as studies on surveillance of molecular markers of anti-malarial drug resistance.

In this context, the study presented here has shown that archived RDTs used for malaria diagnosis in routine practice can be used as an alternative source of DNA for detection of malaria parasites and other molecular analyses. The RDTs can also be used as source of DNA as part of QC testing of RDTs instead of microscopic examination of Giemsa-stained blood smears, by comparing RTD results produced by health workers with those obtained in the laboratory based on detection of malaria parasite by PCR. Thus, after deployment of RDTs in the field, archived RDTs can also be used for QC of RDTs in addition to using Giemsa-stained blood smears, particularly in health facilities without capacity for performing malaria diagnosis by microscopy or lacking the skills and experience to prepare good blood smears as recently shown in Eastern Tanzania by McMorrow and colleagues [17,25].

Storage of RDTs (that were prepared in the laboratory) for one month either with or without silica gel as preservatives did not affect the quality of extracted DNA as determined by the detection limit of PCR in the diluted samples and band intensity of amplified products in comparison to known standard DNA samples which were used as control. Similarly, field samples stored at room temperature without preservatives for over 3 months were equally amplified. However, the impact of long term storage of RDTs beyond this duration on the quantity and quality of DNA is not clearly known, particularly if they are stored at room temperature without preservatives and under high ambient temperature, and humidity (as in most parts of Tanzania) which support growth of moulds that can degrade the parasite DNA. The period of three months or more shown in this and a previous study [23] should be sufficient to have the RDTs delivered to the laboratory even from the most remotely located parts of Tanzania.

Both types of PfHRP-2 based RDTs (ParaHIT^®^f and Paracheck Pf^®^) examined provided DNA of sufficient quality with similar amplification success although the quantity of DNA was not assessed. This is in concordance to a previous study which showed that the type/brand of RDTs did not affect the quality of extracted DNA [23]. Furthermore, the technique proved useful in examining conflicting diagnostic outcomes between microscopy and RDTs. RDT false positive samples showed similar results between PCR and microscopy indicating that most of the samples that were microscopically negative but positive by RDTs were actually negative. False positive results by PfHRP-2 based RDTs are commonly observed due to persistence of the antigens for over 30 days after clearance of parasites following treatment [37]. Among the 24 individuals with false positive RDT results, 14 had taken anti-malarials within seven days before the study; indicating that they may still had persistent PfHRP-2 antigens in their blood. PCR analysis of false negative samples showed conflicting results regarding a subset of samples (11 samples) initially identified by microscopy as positive (but negative by RDTs) and found to be negative by PCR. This could be due to low parasite density since all of these samples had < 100 asexual parasites/μl which is the lowest detection limit of most of the RDTs and also within the lower detection limit of microscopy [13].

## Conclusion

This study showed that DNA can be extracted from routinely used and archived RDTs, and successfully amplified by PCR; thus providing an alternative source of DNA for detection of malaria parasites and possibly other molecular analyses such as surveillance of parasite resistance to anti-malarial drugs and monitoring of insidious parasite reservoirs in different interventions, such as malaria elimination programmes. Furthermore, the RDTs can be used for QC testing of RDTs deployed in the field for malaria diagnosis based on comparing the results obtained by PCR genotyping of DNA extracted from archived RDTs with the test results produced by health workers, particularly from remotely located health facilities without the capacity of microscopic examination of blood smears. Thus, availability of archived RDTs after the tests are deployed in all health facilities in Tanzania as planned by the Ministry of Health and Social Welfare will provide a good source of parasite DNA for laboratory studies.

## Abbreviations

ACT: artemisinin combination therapy; DNA: deoxyribonucleic acid; pLDH: Plasmodium lactate dehydrogenase; NIMR: National Institute for Medical Research; NMCP: National Malaria Control Programme; PCR: polymerase chain reaction; Pf: *Plasmodium falciparum; *PfHRP-2: *Plasmodium falciparum *histidine rich protein 2; RDTs: Rapid diagnostic tests; WBCs: white blood cells; WHO: World Health Organisation; μl: microlitre.

## Competing interests

The authors declare that they have no competing interests.

## Authors' contributions

DSI, MA conceived of the study, designed the experiments, supervised the laboratory analyses and wrote the manuscript. NN, OP and LV participated in study design and supplied archived RDTs while SL and RAM conducted the laboratory analyses. MML and ICB participated in study design and supervised the study. All authors read and approved the manuscript.
